# Transcriptomic analyses identify albino-associated genes of a novel albino tea germplasm ‘Huabai 1’

**DOI:** 10.1038/s41438-018-0053-y

**Published:** 2018-10-01

**Authors:** Qingping Ma, Huan Li, Zhongwei Zou, Emmanuel Arkorful, Qianru Lv, Qiongqiong Zhou, Xuan Chen, Kang Sun, Xinghui Li

**Affiliations:** 1 College of Horticulture, Nanjing Agricultural University, Weigang No.1, 210095 Nanjing, China; 2 Department of Plant Science, University of Manitoba, 222 Agriculture Building, Winnipeg, Manitoba R3T 2N2 Canada

## Abstract

Albinism in shoots of tea plants is a common phenotypic expression which gives the tea infusion a pleasant umami taste. A novel natural albino mutant tea germplasm containing high amino acids content was found and named as ‘Huabai 1’. ‘Huabai 1’ has white jade tender shoots under low temperature and turns green with increased temperature. In order to understand the molecular mechanism of color change in leaf of ‘Huabai 1’, transcriptome analysis was performed to identify albino-associated differentially expressed genes (DEGs). A total of 483 DEGs were identified from white shoots of ‘Huabai 1’ compared to its green shoots. There were 15 DEGs identified to be involved in phenylpropanoid biosynthesis, which account for the majority of characterized DEGs. The metabolites related to phenylpropanoid biosynthesis revealed similar expression pattern of DEGs. Furthermore, metabolic pathways such as ubiquonone, tyrosine, and flavonoid biosynthesis associated with phenylpropanoid biosynthesis could also contribute to the color change in ‘Huabai 1’ tender shoots. Protein–protein interaction analysis revealed a hub protein NEDD8 (CSA009575) which interacted with many regulated genes in spliceosome, nitrogen metabolism, phenylpropanoid biosynthesis, and other pathways. In conclusion, the findings in this study indicate that the color change of ‘Huabai 1’ tender shoots is a combined effect of phenylpropanoid biosynthesis pathway and other metabolic pathways including flavonoid biosynthesis in tea plants. Chlorophyll biosynthesis-related genes LHCII and SGR may also play some roles in color change of ‘Huabai 1’.

## Tea plants: Controlling color and flavor

Researchers have identified genes and related metabolic processes in tea plants that control the lack of green color known as albinism, which gives the tea a savory “umami” taste. The scientists, led by Xinghui Li at Nanjing Agricultural University in China, investigated genes associated with color changes in tender shoots of a novel albino variety of tea plant known as Huabai 1. The transition from white to green shoots is a temperature-dependent process associated with differences in the activity of 483 identified genes. Control of the color change is also linked to alterations in chemical reaction pathways involving a variety of plant compounds, including phenylpropanoids, flavonoids and the green chlorophylls most commonly associated with plant color. The insights may help plant breeders to create new varieties of teas with highly prized desirable flavors

## Introduction

Leaf albinism is a common phenomenon which usually occurs in green plants as a result of chlorophyll deficiency^1–3^. In general, leaf albinism is an abnormal phenotypic expression, and is usually considered to be an undesirable trait in most crop breeding programs because it can cause several unfavorable outcomes such as yield loss and susceptibility to insects and pathogen attack^4,5^. However, in tea plants (*Camellia sinensis*), albinism is of great importance since it produces beneficial metabolites such as amino acids and catechins^6^. Studies have, therefore, shown that lower catechin and higher amino acid contents in albino tea cultivars account for its good flavor^7,8^.

Plant leaf albinism is influenced by various biochemical and environmental factors; however, its molecular mechanisms remain unclear. For instance, carotenoid compounds have been found to be involved in leaf color formation^9^, and suppression of *phytoene desaturase gene* resulted in leaf albinism in tobacco^10^. A number of studies have been conducted to uncover the different molecular mechanisms of leaf albinism in different albino tea cultivars. In ‘Baiye 1’, the leaves remain white under low temperature and turn green with increased temperature. The significant changes in metabolites involved in carbon fixation, phenylpropanoid and flavonoid biosynthesis result in leaf albinism in ‘Baiye 1’^11^. In addition, carbohydrate and energy metabolism, chloroplast biogenesis, and metabolic pathways based on transcriptional and proteomic levels were identified to elucidate the mechanism of color change in the albino tea cultivar ‘Baiye 1’^12^. In addition to ‘Baiye 1’, a light sensitive albino tea cultivar ‘Huangjinya’, whose leaf color shows yellow when exposed to light and turns green after shading, is mainly determined by the combined effects of flavonoid and carotenoid biosynthesis^13^.

The albino tea cultivar ‘Baiye 1’ is the mostly studied and the broadest cultivated because of its large economic value of albino tender shoots and high amino acids. Now, a novel albino tea germplasm, which possesses stable albino phenotype in the offspring by either seeds or asexual reproduction, has been identified and named ‘Huabai 1’. ‘Huabai 1’ shows pure white tender shoots, including white mesophyll and white vein. Dried tea made from albino tender shoots of ‘Huabai 1’ is beautiful, and the tea infusion tastes delicious. Dried tea of ‘Huabai 1’ contains high amino acids (84 mg/g) and tea polyphenol (177 mg/g) which contribute to the umami taste of tea infusion and high antioxidative effect, respectively (unpublished data). In addition, the albino period of ‘Huabai 1’ with economic value is longer by 50% than ‘Baiye 1’. Therefore, ‘Huabai 1’ is an alternative and ideal albino tea germplasm to consider in future breeding programs and plantations.

In order to explore the mechanism of shoot albinism in ‘Huabai 1’, transcriptome analysis of the white and green color shoots of ‘Huabai 1’ was conducted. The main differentially expressed genes (DEGs) and proteins identified in the phenylpropanoid biosynthesis and other metabolic pathways including flavonoid biosynthesis will facilitate the understanding of the mechanism involved in plant leaf albinism and albino tea plant breeding.

## Materials and methods

### Plant materials

Two-year-old ‘Huabai 1’ cuttings were cultivated in the experimental field of Nanjing Agricultural University (Nanjing, China). White tender shoots under low temperature (15/7 °C (day/night)) and green tender shoots under high temperature(30/19 °C (day/night)) were harvested and quickly frozen in liquid nitrogen and stored at −80 °C for RNA extraction and subsequent analysis of metabolites.

### RNA extraction, cDNA library preparation, and sequencing

Total RNA was extracted using EASYspin Plus Plant RNA Extraction Kit (Aidlab Biotech, Beijing, China) according to the manufacture’s instruction. The quality and concentration of extracted RNA was assessed using Agilent 2100 Bio-analyzer (Agilent, USA) and NanoDrop ND-1000 spectrophotometer (NanoDrop, USA), respectively.

A total of 3 μg mixed RNA of each sample was used for cDNA library construction. Sequencing libraries were prepared using NEBNext®Ultra™ RNA Library Prep Kit for Illumina® (NEB, MA, USA) following manufacturer’s instruction. Briefly, the mRNA was purified using Oligo (dT). The purified mRNA was fragmented using NEBNext First Strand Synthesis Reaction Buffer. First strand cDNA was then synthesized using random hexamer primer, and then the second strand was synthesized using RNaseH, DNA Polymerase I, and dNTPs. After adenylation of 3′ end and purification of the cDNA library, polymerase chain reaction (PCR) was performed using Phusion High-Fidelity DNA polymerase. Finally, the PCR products were purified using AMPure XP system (Beckman Coulter, Indianapolis, IN, USA) and the library quality was assessed using the Agilent 2100 Bioanalyzer. After clustering with TruSeq PE Cluster Kit v3-cBot-HS (Illumia), the generated cDNA library was sequenced on Illumina HiSeq^TM^ 2500 platform (Biomarker Biotech, Beijing, China) and paired-end reads were generated. Three biological replicates were conducted for each sample.

### Quality control and transcriptome analysis

The clean data were produced by removing low quality reads, adapter, and reads with ploy-A. The clean reads were aligned to the tea plant genome^14^ (http://www.plantkingdomgdb.com/tea_tree/) by TopHat2^15^. According to the reference genome, the unannotated genes were identified using Cufflinks software^16^. These genes were annotated by blast to NCBI non-redundant protein sequences (Nr) searching^17^, Pfam identification^18^, and to blast with Clusters of Orthologous Groups of proteins (KOG/COG)^19,20^, Swiss-Prot^21^, Kyoto Encyclopedia of Genes and Genomes (KEGG)^22^ and Gene Ontology (GO)^23^.

### Differential expression analysis

Fragments Per Kilobase of transcript per Million fragments (FPKM) was used for the evaluation of expression of the transcripts^24^. Biological variability of the samples were assessed by Pearson’s Correlation Coefficient of which *r*^2^ close to 1 indicated a high correlation of the replicates^25^. DESeq was applied for differential expression analyses of genes^26^. Expressions, generated through Benjamini–Hochberg adjustment of *p*-value, with fold change ≥2 and false discovery rate (FDR) <0.01 were considered as DEGs.

### Quantitative real-time PCR (qRT-PCR) verification analysis

To validate the reliability of RNA-Seq data, qRT-PCR was performed. The first strand cDNA was synthesized using the RevertAid First Strand cDNA Synthesis Kit (Thermo Scientific, USA). The qRT-PCR was performed in 20 μL reaction volume containing 1 μL of 100 ng cDNA, 0.5 μL each of 10 μM forward and reverse primers, 10 μL SYBR Premix Ex Taq II (Takara, Japan) and filled with 8 μL ddH_2_O. The primers of candidate DEGs used for qRT-PCR are listed in Table 1. The qPCR was conducted on Roche 480 II system (Switzerland) with the following thermos cycling: 95 °C for 30 s, 95 °C for 5 s, and 60 °C for 30 s for 40 cycles. All the samples were performed in three biological replicates. The glyceraldehyde-3-phosphate dehydrogenase gene (*GAPDH*), *actin* (*ACTIN*), and 18S ribosomal RNA (*18S rRNA*) were used as the reference genes^27^. The relative expressions of investigated genes were calculated using 2^−ΔΔCT^ method^28^.

**Table 1:** The primers used for quantitative RT-PCR verification

Gene ID	Gene name	Forward (5′-3′)	Reverse (5′-3′)	Product size (bp)
CSA016595	Galactinol synthase 2	GGTCACGCTTCCTACTTCAT	GCATGTTCTCTTCCTGTCCA	209
CSA031871	Thioredoxin-like protein CXXS1	GTCCATTTTACTGCTTCTTG	CCATCCTTGTTGCTACCTCC	136
CSA021228	Transparent testa 12	GCATCCAAATCTACTACTAC	GGTGTTGTGTTTTGCTGATG	141
CSA030945	Plant cadmium resistance 2	GCAGAGATTGTTGACGAAGG	CTAAACAATCACCACAAGGG	163
CSA027637	Reticuline oxidase-like protein	TCACACATCCAAGCAGCCAT	GGTCTCGTCTTCTATGCTGA	168
CSA011590	KH domain-containing protein	GAAGAACCAATAGAGGACCC	ACATTACTGGAGAAACACAC	178
CSA013206	Putative disease resistance protein RGA1	GTCATTGTGTGGGAGGAGAT	CTTGAGATGGTATGTGGAAT	184
CSA002486	Thaumatin-like protein	CTGACATAGTTGGCGAGTGC	CATCTGGGCACCTATCCTTG	185
CSA011958	Coffea canephora DH200	ATGTCGTCTCCAACTCCCTC	ATCAAAACCATTACAGGGCT	155
*GAPDH*	Glyceraldehyde-3-phosphate dehydrogenase	GTTTGGCGTCGTTGAGGGTC	GGCAGCACCTTACCAACAG	164
*ACTIN*	ACTIN	GAACCCGAAGGCGAATAGG	ACCATCACCAGAATCCAAGAC	145
*18S rRNA*	18S rRNA	TCTGCCCGTTGCTCTGATG	TCACCCGTCACCACCATAG	134

### Detection of the metabolites on phenylpropanoid biosynthesis

Metabolites were extracted from 100 mg tea shoots using 1.0 mL 70% methanol containing 0.1 mg/L Lidocaine and incubated at 4 °C overnight. After centrifugation at 10,000×*g* for 10 min, the supernatant was filtered with 0.22 μm pore size membrane for further liquid chromatography-tandem mass spectrometry analysis (LC-MS). Five microliters of each sample was detected using the Ultra Performance Liquid Chromatography (UPLC, Shim-pack UFLC SHIMADZU CBM20A, http://www.shimadzu.com.cn/) with Waters ACQUITY UPLC HSS T3 C18 1.8 µm column (2.1 mm×100 mm). Different ratios of water/acetonitrile were used as eluting agent in the following gradients: 95:5 V/V at 0 min, followed by 5:95 V/V at 11.0 min, 5:95 V/V at 12.0 min, 95:5 V/V at 12.1 min, and 95:5 V/V at 15.0 min. The flow rate was 0.4 mL/min, and the column temperature was 40 °C. Isolated samples were analyzed using the Applied Biosystems 4500 QTRAP MS system (http://www.appliedbiosystems.com.cn/). The peak area was recorded and normalized to characterize the metabolite differences between green and white ‘Huabai 1’ tender shoots.

### Statistical analysis

Statistical analysis was performed using Microsoft Excel 2016 and GraphPad Prism 5.0 (https://www.graphpad.com/scientific-software/prism/). One-way ANOVA was used for significant difference analysis, and *P* < 0.05 was considered to be significantly different.

## Results

### Phenotypic characteristics of ‘Huabai 1’

**Fig. 1:**
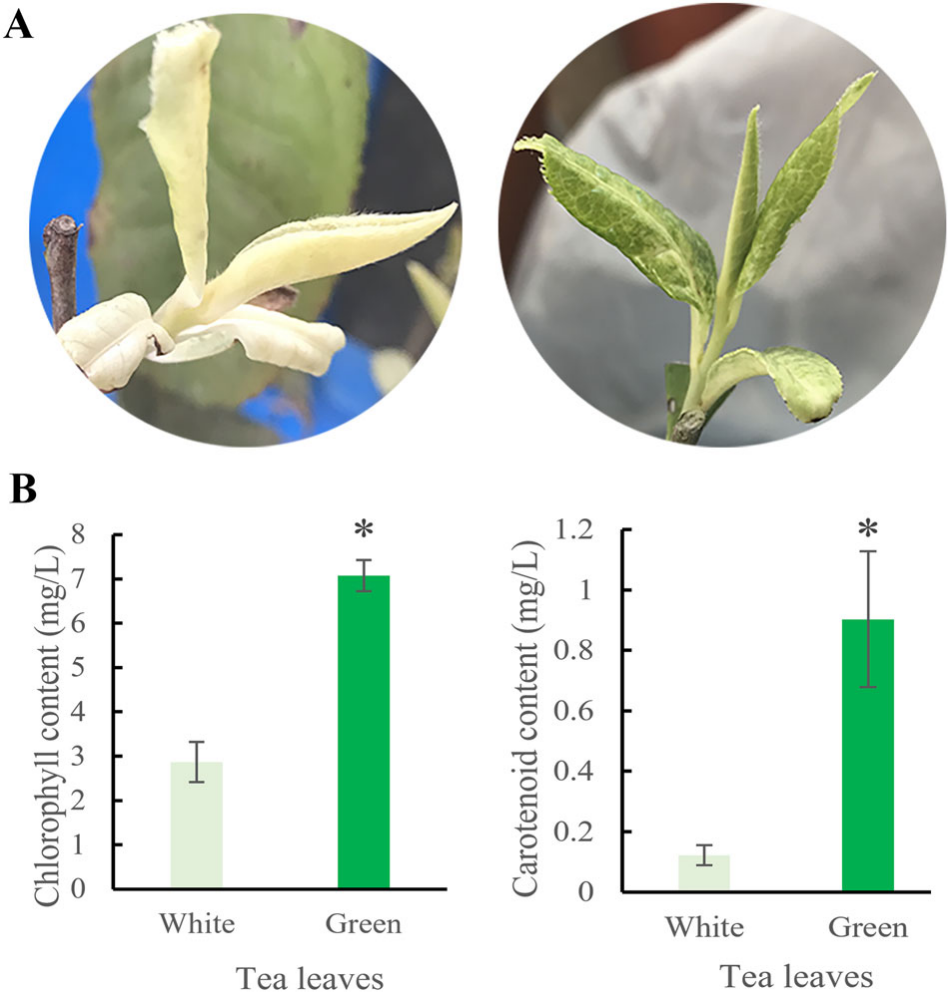
The phenotype and chlorophyll content of white and green ‘Huabai 1’ leaves. **a** leaf color of ‘Huabai 1’ new shoots. **b** Chlorophyll and carotenoid content of ‘Huabai 1’ new shoots; * represents significant difference

Under low temperatures of 15/7 °C (day/night), tender shoots of ‘Huabai 1’ were pure white. However, at high temperatures of 30/19 °C (day/night), shoots turned green, while leaf veins remained white (Fig. 1a). The chlorophyll and carotenoid contents of the green shoots were significantly higher than in albino shoots (Figure 1B and Table S1).

### DEGs identification

**Fig. 2:**
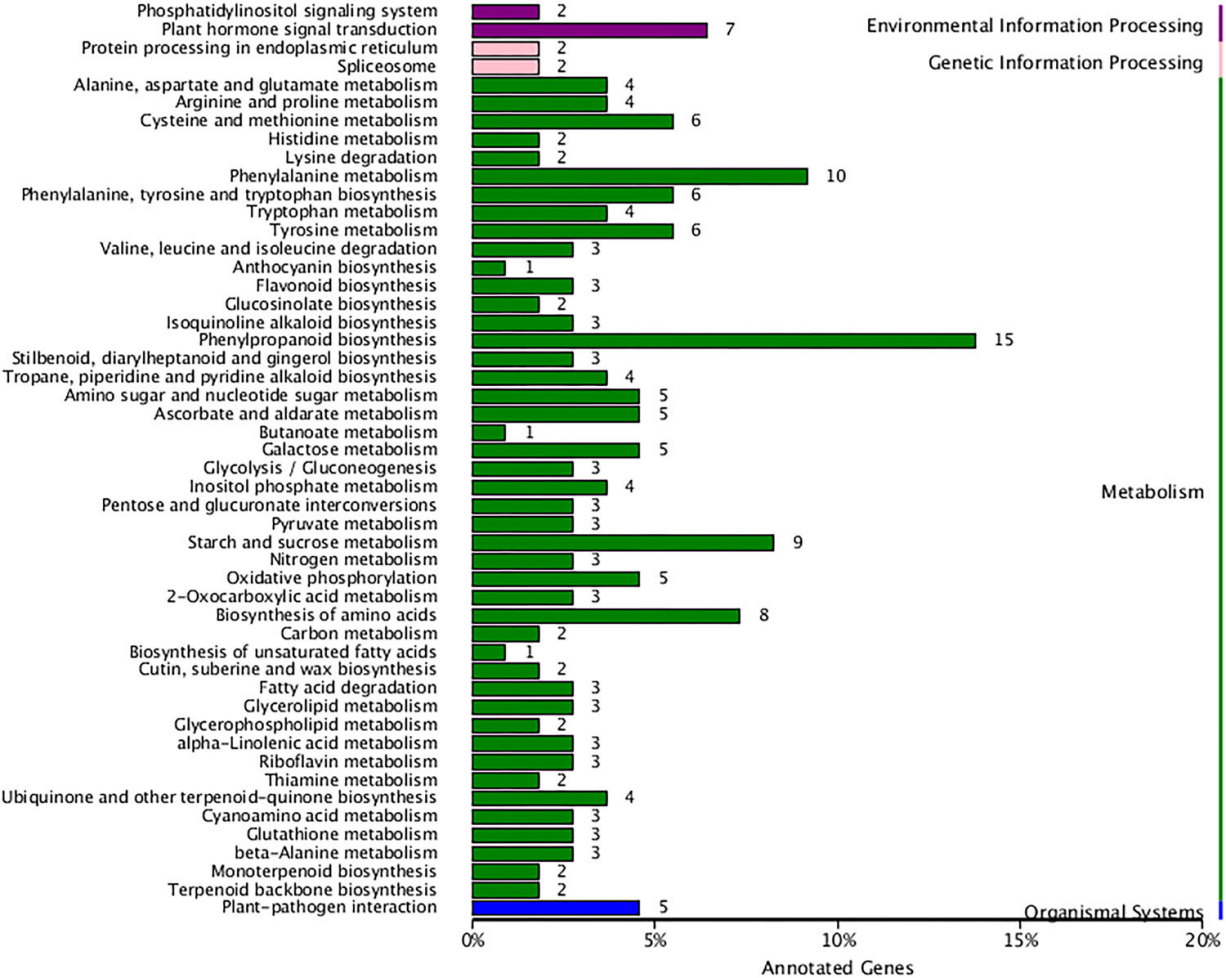
**Differentially expressed genes enriched on different KEGG pathways.**

Q30 of the raw data ranged from 89.60 to 91.02% indicating a high-quality reads worthy of further analysis. More than 60% (63.93–65.24) reads of each sample mapped with tea genome (Table 2). The clean reads of the samples in the present study were deposited in the Sequence Read Archive (SRA) of NCBI database, and accession number SRP126084 was obtained. After comparing the green shoots to white shoots of ‘Huabai 1’, a total of 483 DEGs were identified. Among them, 471 genes were exclusively annotated with 182, 331, 170, 203, 471, 395, and 389 annotated genes from COG, GO, KEGG, KOG, NR, Swiss-Prot, and TrEMBL databases, respectively. KEGG pathway analysis revealed that most of the DEGs were enriched in metabolism pathway. There were 15 DEGs identified in the phenylpropanoid biosynthesis pathway, followed by phenylalanine metabolism (10 DEGs), starch and sucrose metabolism (9 DEGs), and biosynthesis of amino acids (8 DEGs) (Fig. 2).

**Table 2:** The quality of the transcriptome of white and green ‘Huabai 1’ leaves

Samples	GC content	%≥Q30	Total reads	Mapped reads
White-1	44.56%	89.79%	49,281,276	31,503,245 (63.93%)
White-2	45.31%	89.60%	47,142,112	30,423,001 (64.53%)
White-3	45.40%	89.77%	44,300,068	28,726,569 (64.85%)
Green-1	45.23%	91.02%	43,114,402	28,126,192 (65.24%)
Green-2	45.02%	90.14%	48,858,098	31,515,833 (64.50%)
Green-3	45.10%	90.42%	45,656,596	29,720,851 (65.10%)

**Fig. 3:**
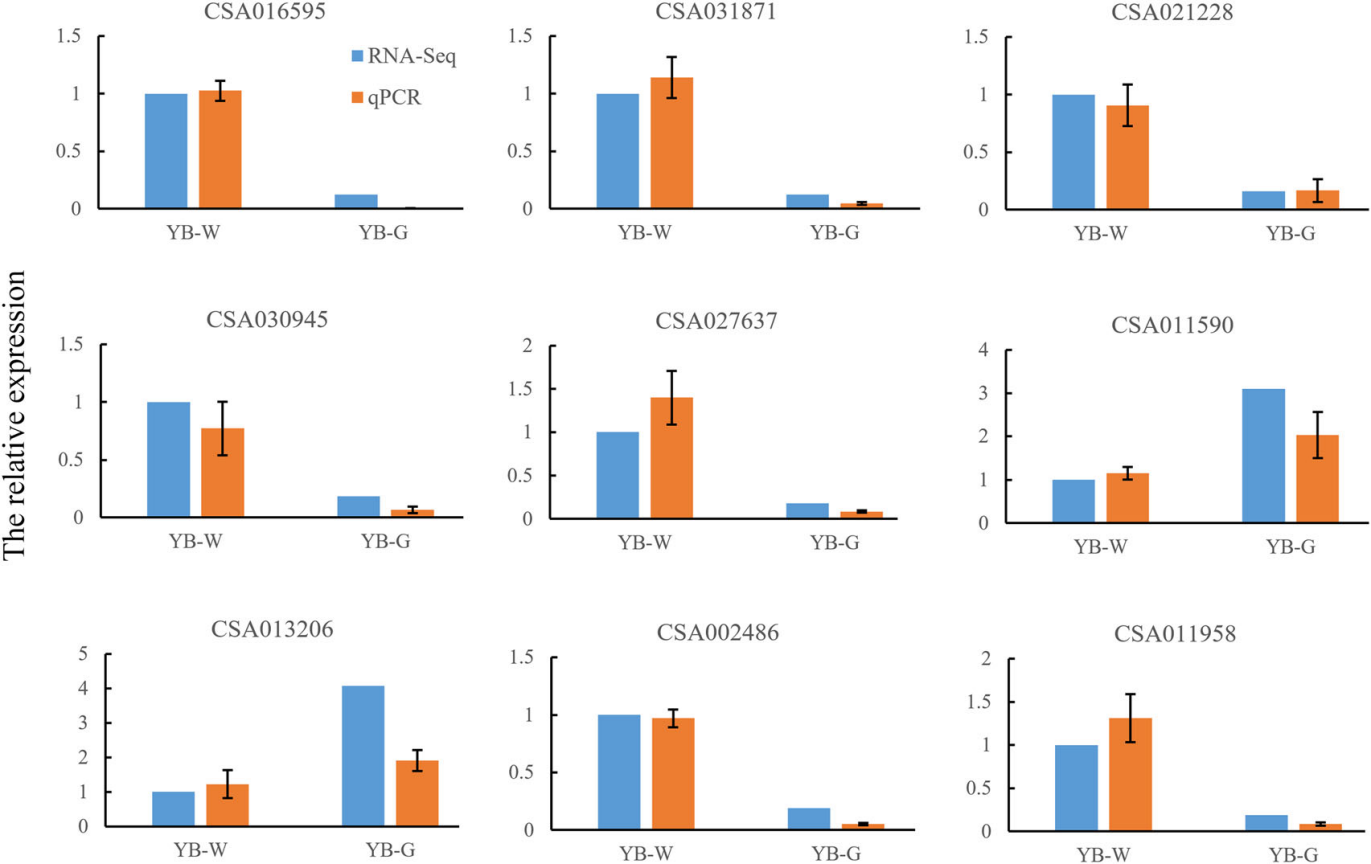
Quantitative RT-PCR verification for randomly selected differentially expressed genes. RNA-Seq and qRT-PCR data were displayed as fold changes

In order to verify the reliability of the DEG expression, 9 DEGs were randomly selected for qRT-PCR analysis. The results indicated similar expression patterns in transcript abundance analysis by RNA-Seq and qRT-PCR (Fig. 3).

### DEGs and metabolites on phenylpropanoid biosynthesis

**Fig. 4:**
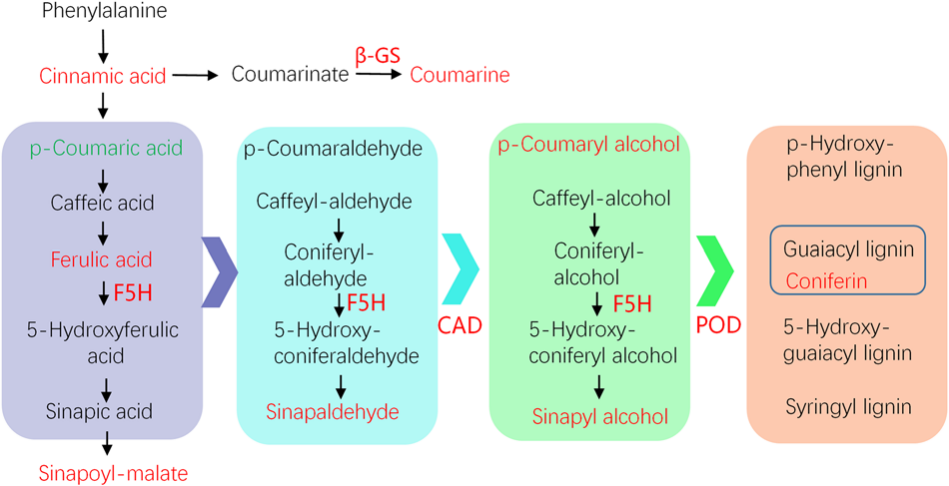
The differentially expressed genes and metabolites on phenylpropanoid biosynthesis. Red and green, respectively, mean up- and downregulation in white leaves compared to green leaves of ‘Huabai 1’

The expression of the genes involved in phenylpropanoid biosynthesis of white tender shoots showed significant higher levels than that of green shoots, especially for the genes related to deoxygenation and hydrogenation reduction reactions. As shown in Fig. 4, *ferulate-5-hydroxylase* (*F5H*), which encoded the cytochrome P450 isoform in association with syringyl lignin precursor hydroxylation, expressed significantly higher in white tender shoots than in green shoots. The expressions of *cinnamyl-alcohol dehydrogenase* (*CAD*) catalyzing the alcoholization, and *Peroxidase* (*POD*) in lignin synthesis, were also higher in white tender shoots. The relative changes of the metabolites downstream of phenylprponoid biosynthesis including sinapoyl-malate, sinapaldehyde, and sinapyl alcohol were consistent with the expressions of the corresponding genes. In addition, *beta-glucosidase* (*β-GS*) directed deglycosylation of β-D-glucosyl-Coumarate, and led to accumulation of coumarine (Fig. 4).

### Other pathways related to phenylpropanoid biosynthesis

**Fig. 5:**
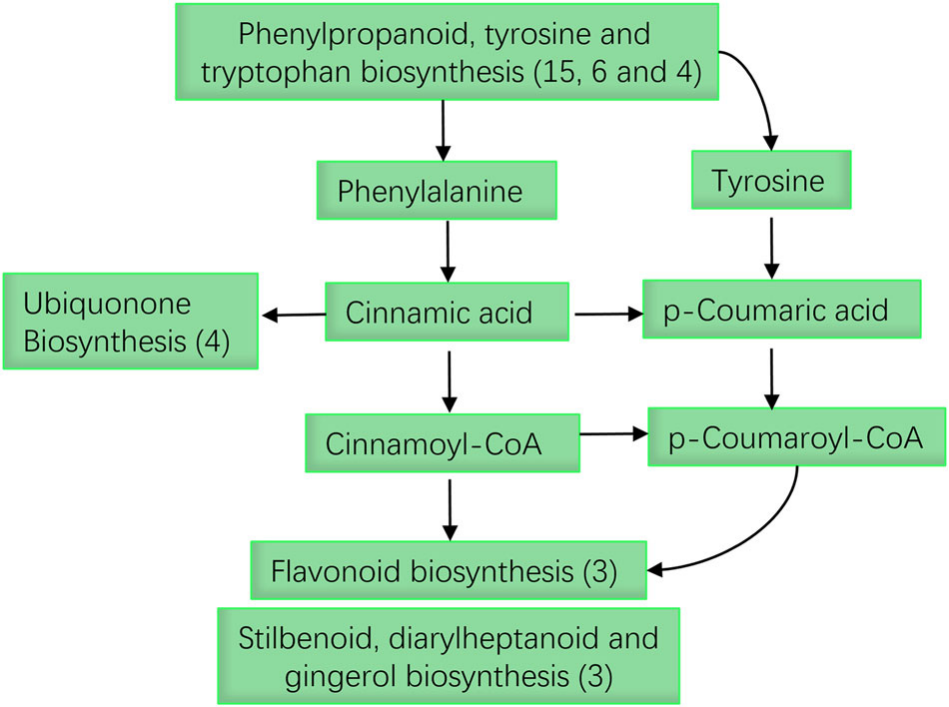
**Phenylpropanoid biosynthesis-associated metabolic pathways in responding to color change of ‘Huabai 1’.**

In addition to phenylpropanoid biosynthesis, several other metabolic pathways were also enriched with DEGs, including six in tyrosine, four in tryptophan, four in ubiquonone, three in flavonoid biosynthesis, and three in ‘stilbenoid, diaryheptanoid, and gingerol biosynthesis’. This result, therefore, suggests that leaf color change in ‘Huabai 1’ is a combined effect of the above-mentioned phenylpropanoid biosynthesis associated pathways (Fig. 5).

### Chlorophyll biosynthesis-related genes

Chlorophyll deficiency is the direct expression of albino tea plants. Among 471 DEGs, two genes contributing to chlorophyll biosynthesis were identified: *light-harvesting complex II chlorophyll a/b binding protein* (*LHCII*, CSA035910) and *STAY-GREEN* (*SGR*, CSA024979). The expression of *LHCII* was higher in green tea shoots (2 folds) but *SGR* was higher in albino tea shoots (5.76 folds).

### Protein–protein interaction analysis

**Fig. 6:**
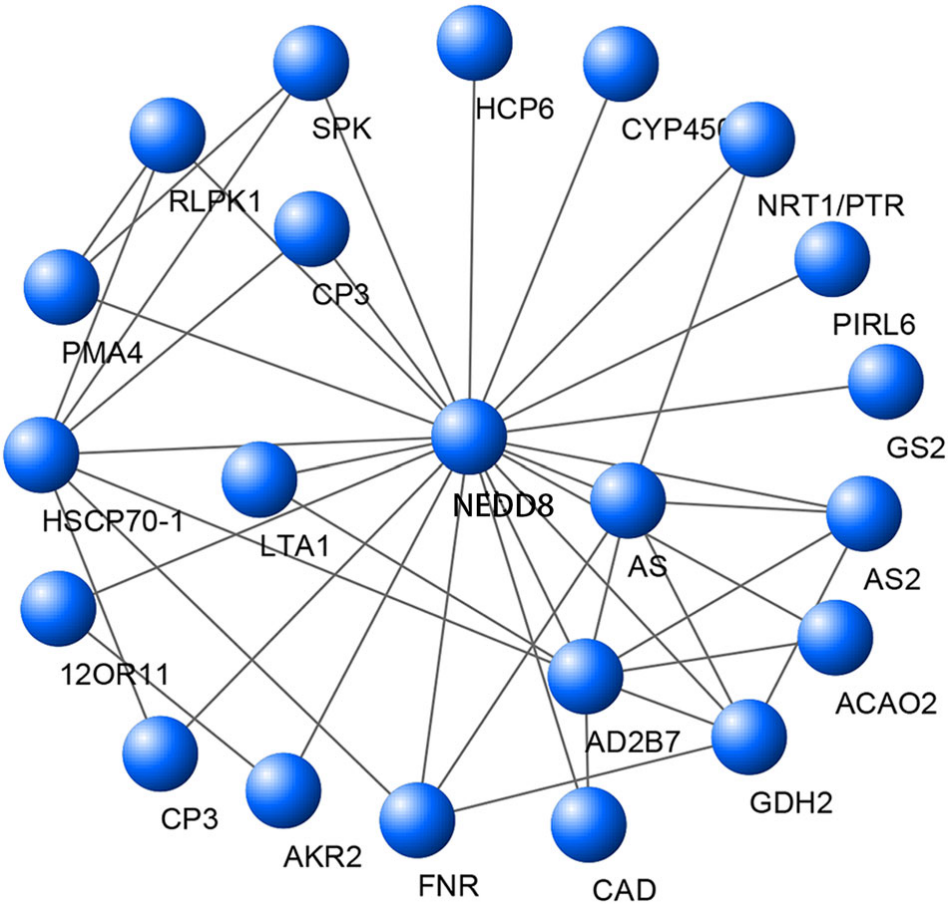
Protein–protein interaction network analysis based on the differentially expressed genes. The definition and description of the proteins in this network are shown in Table S1

Functional partnerships and interactions occur between proteins, and such interactions are usually at the core of cellular processing. In the present study, protein interaction networks revealed the presence of a hub protein, ‘neural precursor cell expressed developmentally downregulated 8’ (NEDD8), in albino leaves. This protein is also known as Related To Ubiquitin (RUB). It was found to have correlations with proteins of many metabolic pathways, of which the major ones included heat shock 70 kDa protein in spliceosome, ferredoxin-nitrite reductase and glutamate dehydrogenase in nitrogen metabolism, CAD in phenylpropanoid biosynthesis, and galactinol synthase in galactose metabolism (Fig. 6 and Table S2).

## Discussion

Albinism in tender shoots is a desirable trait in most tea plant breeding programs due to its high amino acid and low catechins contents. ‘Baiye 1’ is not only the most studied albino tea cultivar in tea breeding program, but also the widely cultivated cultivar in most tea plantations^29^. However, the defects of shortened and unstable albinism are more pronounced in this cultivar. Thus, the ‘Huabai 1’ with pure white tender shoots and long albinism period is an alternative and ideal germplasm. In this study, transcriptome and metabolite analysis were employed to investigate the mechanism of albinism in ‘Huabai 1’ tea plants. A total of 15 DEGs were identified to be involved in phenylpropanoid biosynthesis. These DEGs were found to be the most influential factors that contributed to color changes of leaves in ‘Huabai 1’. Meanwhile, phenylpropanoid biosynthesis pathways were also observed to play a considerable role in color change in ‘Huabai 1’. In addition, a hub protein, NEDD8, was characterized through protein–protein interaction analysis. This protein has correlations to several metabolic pathways including spliceosome, nitrogen metabolism, and phenylpropanoid biosynthesis.

The phenylpropanoid metabolism generates a large number of secondary metabolites through many enzymes, like ligases, oxygenases, oxidoreductases, and transferases^30^. Lignin biosynthesis is a major branch of phenylpropanoid biosynthesis and produces lignin polymers^31^. Lignin is exclusively based on phenylpropanoid units derived from the oxidative polymerization of cinnamoyl alcohol derivatives. Cinnamyl alcohol dehydrogenase catalyzes the final step in monolignol biosynthesis, transferring the –aldehydes to –alcohols. Disruption of the *CAD* leads to altered lignification and promotes saccharification in *Brachypodium distachyon*^32^. As well, *CAD* can change the lignin structure and loss of function of *CAD* would lead to temperature sensitive defects^33^. In the present study, *CAD* of ‘Huabai 1’ was highly expressed in albino tea tender shoots. This observation can be explained by the mechanism involved in change in lignin structure as a result of loss of function of *CAD*, and its resultant effect on leaf coloration. The results in the present study, therefore, suggest that albinism in ‘Huabai 1’ is a cold sensitive phenotypic expression. This is due to higher expression of *CAD* in albino tea tender shoots than in green tender shoots. However, leaf albinism in ‘Huabai 1’ restored to normalcy with increased temperature.

With the exception of *CAD*, *F5H* is another dominant gene involved in lignin biosynthesis, which could control the ratio of syringyl (S)/guaiacyl (G) lignin. Downregulation of *F5H* produced more lignin with G units, whereas upregulation of *F5H* resulted in lignin with S units^34^. In white ‘Huabai 1’ tender shoots, the expression of *F5H* gene was higher than in green tender shoots. Meanwhile, the S-lignin precursors, including sinapoyl-malate, sinapaldehyde, and sinapyl alcohol in white tender shoots were also higher than in green tender shoots (Fig. 4). These results indicate a positive correlation between *F5H* and S-lignins precursors in tea plants. Therefore, the higher the *F5H* expressed, the more the S-lignins precursor content were produced. S-lignins were derived from the oxidative polymerization of sinapyl alcohol under POD catalyzation. Although higher sinapyl alcohol content and *POD* expression were observed in white ‘Huabai 1’ tender shoots, there was no significant changes in the S-lignin found. Therefore, it suggests that S-lignin may not be the unique result of sinapyl alcohol polymerization. This suggests that sinapyl alcohol can produce other derivatives apart from S-lignin. Tian et al.^35^ have identified four new sinapyl alcohol derivatives dichrocephols A–D from Lipo-soluble part of *Dichrocephala benthamii*. These derivatives may also exist in tea tender shoots.

**Fig. 7:**
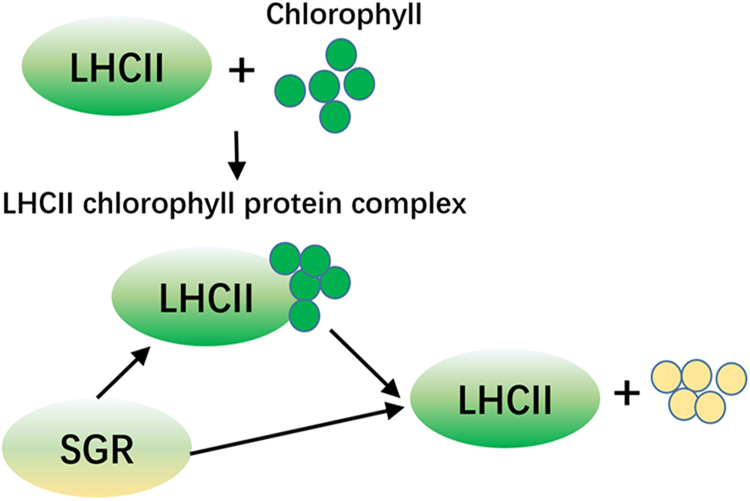
**The model of role of LHCII and SGR proteins on chlorophyll catalytic process.**

According to the annotation of the DEGs, two chlorophyll catalytic process related genes *LHCII* and *SGR* were identified. LHCII protein embedded in chloroplast membranes and the aggregation of LHCII chlorophyll protein complex could control the light harvesting function of chloroplast membranes^36^. However, *SGR* expressed in senescing plastids and was required for the degradation of chlorophyll^37^. In addition, SGR could interact with LHCII and led to breakdown of LHCII-located chlorophyll (as the model shown in Fig. 7)^37^. Therefore, in ‘Huabai 1’ albino shoots, the higher expression of *SGR* and lower expression of *LHCII* suggest that the LHCII chlorophyll biosynthesis was inhibited and the breakdown of chlorophyll was accelerated. This may be part of cause of the color change in ‘Huabai 1’ shoots.

In this study, the color change in ‘Huabai 1’ was based on a network with the core gene of *NEDD8*. Like ubiquitin, *NEDD8* is conjugated to the substrate protein through the isopeptide bond between C-terminal glycine and a lysine residue of the target protein (neddylation)^38^. *NEDD8* modification is a post-translational modification of the cullin subunits of cullin-RING E3 ubiquitin ligases. Boh et al. reported that neddylation induced a conformation change of cullin RING ligases domains via structural analysis in vitro, and then promotes ubiquitin transfer onto the substrate^39^. In plants, the NEDD8‐conjugating enzyme mutation resulted in lethal phenotype, indicating that the NEDD8 conjugation pathway plays an important role in plant growth and development^39^. In *Mirabilis jalapa*, petroleum stress induced accumulation of a number of proteins including NEDD8^40^. Therefore, the expression changes of NEDD8 and related proteins in ‘Huabai 1’ tender shoots would contribute to the leaf color change.

In conclusion, phenylpropanoid biosynthesis contributed to the color change in ‘Huabai 1’ tender shoots. Chlorophyll biosynthesis-related genes (*LHCII* and *SGR*) and a protein network with the core of NEDD8 may also be an influencing factor in leaf color change in ‘Huabai 1’.

## Electronic supplementary material

Table S1

Table S2

## References

[1] Wu Z (2007). A chlorophyll-deficient rice mutant with impaired chlorophyllide esterification in chlorophyll biosynthesis. Plant Physiol..

[2] Campbell BW (2015). Identical substitutions in magnesium chelatase paralogs result in chlorophyll-deficient soybean mutants. G3.

[3] Zhu L (2014). Genetic characterisation and fine mapping of a chlorophyll-deficient mutant (BnaC.ygl) in *Brassica napus*. Mol. Breed..

[4] Choi HG (2014). Yield loss and quality degradation of strawberry fruits cultivated under the deficient insolation conditions by shading. Hortic. Environ. Biotech..

[5] Slattery RA (2017). Photosynthesis, light use efficiency, and yield of reduced-chlorophyll soybean mutants in field conditions. Front. Plant Sci..

[6] Feng L (2014). Determination of quality constituents in the young leaves of albino tea cultivars. Food Chem..

[7] Du YY (2006). A study on the chemical composition of albino tea cultivars. J. Hortic. Sci. Biotech..

[8] Wei K (2012). Comparison of catechins and purine alkaloids in albino and normal green tea cultivars (*Camellia sinensis* L.) by HPLC. Food Chem..

[9] Yuan H, Zhang J, Nageswaran D, Li L (2015). Carotenoid metabolism and regulation in horticultural crops. Hortic. Res..

[10] Wang M, Wang G, Ji J (2010). Suppression of the phytoene desaturase gene influence on the organization and function of photosystem II (PSII) and antioxidant enzyme activities in tobacco. Environ. Exp. Bot..

[11] Li CF (2015). Differential Metabolic Profiles during the Albescent Stages of ‘Anji Baicha’ (*Camellia sinensis*). PLoS ONE.

[12] Li Q (2011). Proteomic analysis of young leaves at three developmental stages in an albino tea cultivar. Proteome Sci..

[13] Song L (2017). Molecular link between leaf coloration and gene expression of flavonoid and carotenoid biosynthesis in *Camellia sinensis* cultivar ‘Huangjinya’. Front. Plant Sci..

[14] Xia EH (2017). The tea tree genome provides insights into tea flavor and independent evolution of caffeine biosynthesis. Mol. Plant.

[15] Kim D (2013). TopHat2: accurate alignment of transcriptomes in the presence of insertions, deletions and gene fusions. Genome Biol..

[16] Trapnell C (2010). Transcript assembly and quantification by RNA-Seq reveals unannotated transcripts and isoform switching during cell differentiation. Nat. Biotechnol..

[17] Deng, Y. Y. et al. Integrated nr database in protein annotation system and Its localization. *Computer Engineering* **32**, 71–73 (2006).

[18] Finn RD (2014). Pfam: the protein families database. Nucleic Acids Res..

[19] Tatusov RL, Galperin MY, Natale DA, Koonin EV (2000). The COG database: a tool for genome-scale analysis of protein functions and evolution. Nucleic Acids Res..

[20] Koonin EV (2004). A comprehensive evolutionary classification of proteins encoded in complete eukaryotic genomes. Genome Biol..

[21] Apweiler R (2004). UniProt: the Universal Protein knowledgebase. Nucleic Acids Res..

[22] Kanehisa M (2004). The KEGG resource for deciphering the genome. Nucleic Acids Res..

[23] Ashburner M (2000). Gene ontology: tool for the unification of biology. The Gene Ontology Consortium. Nat. Genet..

[24] Florea L, Song L, Salzberg SL (2013). Thousands of exon skipping events differentiate among splicing patterns in sixteen human tissues. F1000Res..

[25] Schulze SK (2012). SERE: single-parameter quality control and sample comparison for RNA-Seq. BMC Genomics.

[26] Anders S, Huber W (2010). Differential expression analysis for sequence count data. Genome Biol..

[27] Ma QP, Hao S, Chen X, Li XH (2016). Validation of reliability for reference genes under various abiotic stresses in tea plant. Russ. J. Plant Physiol..

[28] Livak KJ, Schmittgen TD (2001). Analysis of relative gene expression data using real-time quantitative PCR and the 2(-Delta Delta C(T)) Method. Methods.

[29] Li CF (2016). Biochemical and transcriptomic analyses reveal different metabolite biosynthesis profiles among three color and developmental stages in ‘Anji Baicha’ (*Camellia sinensis*). BMC Plant Biol..

[30] Vogt T (2010). Phenylpropanoid biosynthesis. Mol. Plant.

[31] Boerjan W, Ralph J, Baucher M (2003). Lignin Biosynthesis. Annu. Rev. Plant. Biol..

[32] Bouvier d’Yvoire M (2013). Disrupting the cinnamyl alcohol dehydrogenase 1 gene (BdCAD1) leads to altered lignification and improved saccharification in *Brachypodium distachyon*. Plant J..

[33] Zhao Q (2013). Loss of function of cinnamyl alcohol dehydrogenase 1 leads to unconventional lignin and a temperature-sensitive growth defect in *Medicago truncatula*. PNAS.

[34] Takeda Y (2017). Regulation of coniferaldehyde 5-hydroxylase expression to modulate cell wall lignin structure in rice. Planta.

[35] Tian X (2013). Sinapyl alcohol derivatives from the lipo-soluble part of *Dichrocephala benthamii* C. B. Clarke. Molecules.

[36] Pascal AA (2005). Molecular basis of photoprotection and control of photosynthetic light-harvesting. Nature.

[37] Sakuraba Y (2012). STAY-GREEN and chlorophyll catabolic enzymes interact at light-harvesting complex II for chlorophyll detoxification during leaf senescence in ara*bidopsis*. Plant Cell.

[38] Schwechheimer, C. & Mergner, J. The NEDD8 modification pathway in plants. *Front. Plant Sci*. **5**, 103 (2014). 10.3389/fpls.2014.00103 PMC3968751 24711811

[39] Boh BK, Smith PG, Hagen T (2011). Neddylation-induced conformational control regulates cullin RING ligase activity in vivo. J. Mol. Biol..

[40] Chen S (2017). Quantitative proteomics analysis reveals the tolerance of *Mirabilis jalapa* L. to petroleum contamination. Environ. Sci. Pollut. Res..

