# Supplementary material for: Transcriptomic analyses identify albino-associated genes of a novel albino tea germplasm ‘Huabai 1’
Source: Hortic Res. 2018 Oct 1;5:54. doi: 10.1038/s41438-018-0053-y (PMC6165850; doi:10.1038/s41438-018-0053-y)
Supplement: Supplementary file 1 — Table S1 [file 41438_2018_53_MOESM1_ESM.docx]

**Table S1 Chlorophyll and carotenoid content of ‘Huabai 1’ new shoots.**

| Content (mg/L) | White | Green |
| --- | --- | --- |
| Chlorophyll | 2.87±0.46b | 7.07±0.35a |
| Carotenoid | 0.12±0.03b | 0.90±0.23a |

Notes: Different letters denote significant difference.
