# Supplementary material for: Transcriptomic analyses identify albino-associated genes of a novel albino tea germplasm ‘Huabai 1’
Source: Hortic Res. 2018 Oct 1;5:54. doi: 10.1038/s41438-018-0053-y (PMC6165850; doi:10.1038/s41438-018-0053-y)
Supplement: Supplementary file 2 — Table S2 [file 41438_2018_53_MOESM2_ESM.docx]

**Table S2 The genes for protein-protein interaction network analysis.**

| Gene ID | Annotation | Abbreviation |
| --- | --- | --- |
| CSA009575 | NEDD8-like protein RUB2 (Precursor) | NEDD8 |
| CSA008397 | ADP,ATP carrier protein 3 | CP3 |
| CSA033635 | ADP,ATP carrier protein 3 | CP3 |
| CSA011618 | Serine/threonine-protein kinase | SPK |
| CSA020217 | Hexose carrier protein HEX6 | HCP6 |
| CSA022028 | Cytochrome P450 86B1 | CYP450 |
| CSA029198 | Protein NRT1/ PTR FAMILY 7.3 | NRT1/ PTR |
| CSA016595 | Galactinol synthase 2 | GS2 |
| CSA035384 | Asparagine synthetase | AS |
| CSA026969 | Acyl-coenzyme A oxidase 2 | ACAO2 |
| CSA011735 | Glutamate dehydrogenase 2 | GDH2 |
| CSA026155 | Cinnamyl-alcohol dehydrogenase | CAD |
| CSA003866 | Ferredoxin--nitrite reductase | FNR |
| CSA034509 | Probable aldo-keto reductase 2 | AKR2 |
| CSA029366 | Putative 12-oxophytodienoate reductase 11 | 12OR11 |
| CSA000963 | Heat shock cognate 70 kDa | HSCP70-1 |
| CSA018666 | Plasma membrane ATPase 4 | PMA4 |
| CSA000646 | Receptor-like protein kinase THESEUS 1 (Precursor) | RLPK1 |
| CSA032513 | Aldehyde dehydrogenase family 2 member B7 | AD2B7 |
| CSA005667 | Adenylosuccinate synthetase 2 | AS2 |
| CSA009942 | Plant intracellular Ras-group-related LRR protein 6 | PIRL6 |
